# Treatment performance and microbial community structure in an aerobic granular sludge sequencing batch reactor amended with diclofenac, erythromycin, and gemfibrozil

**DOI:** 10.3389/frmbi.2023.1242895

**Published:** 2023-09-22

**Authors:** Kylie B. Bodle, Rebecca C. Mueller, Madeline R. Pernat, Catherine M. Kirkland

**Affiliations:** 1 Department of Civil Engineering, Montana State University, Bozeman, MT, United States,; 2 Center for Biofilm Engineering, Montana State University, Bozeman, MT, United States,; 3 United States Department of Agriculture (USDA) Agricultural Research Service, Western Regional Research Center, Albany, CA, United States

**Keywords:** aerobic granular sludge, wastewater treatment, pharmaceuticals and personal care products, emerging contaminants, biodegradation, bioremediation, microbial activity, microbiome

## Abstract

This study characterizes the effects of three commonly detected pharmaceuticals—diclofenac, erythromycin, and gemfibrozil—on aerobic granular sludge. Approximately 150 μg/L of each pharmaceutical was fed in the influent to a sequencing batch reactor for 80 days, and the performance of the test reactor was compared with that of a control reactor. Wastewater treatment efficacy in the test reactor dropped by approximately 30–40%, and ammonia oxidation was particularly inhibited. The relative abundance of active *Rhodocyclaceae, Nitrosomonadaceae*, and *Nitrospiraceae* families declined throughout exposure, likely explaining reductions in wastewater treatment performance. Pharmaceuticals were temporarily removed in the first 12 days of the test via both sorption and degradation; both removal processes declined sharply thereafter. This study demonstrates that aerobic granular sludge may successfully remove pharmaceuticals in the short term, but long-term tests are necessary to confirm if pharmaceutical removal is sustainable.

## 1 Introduction

Pharmaceutical consumption has increased concomitantly with population growth (Bernot et al., 2016). A natural consequence of this is increasing pharmaceutical contamination in the environment, due in part to incomplete metabolism by humans, followed by poor removal by conventional wastewater treatment systems (Xia et al., 2005; Bernot et al., 2016). As such, improved wastewater treatment methods are worthy of investigation, as exposure to pharmaceuticals can cause antibiotic resistance gene proliferation as well as numerous other adverse effects on plants, animals, and microbiota (Kim and Aga, 2007; Bexfield et al., 2019; Świacka et al., 2021).

Aerobic granular sludge (AGS) is an emerging wastewater treatment biotechnology that may be capable of enhancing pharmaceutical removal from wastewater (Zhao et al., 2015; Wang et al., 2018). AGS is highly diverse with populations of nitrifying, denitrifying, and phosphate-accumulating organisms that self-aggregate into spherical biofilms. The gel-like extracellular polymeric substances (EPS) secreted by bacteria in AGS confer protection from toxins and enhance AGS density, resulting in short settling times and high biomass retention (Adav et al., 2008). Furthermore, the EPS in AGS may provide a sorptive medium for organic compound removal (Kong et al., 2015; Kent and Tay, 2019). However, the body of literature on AGS-driven pharmaceutical treatment is limited, and therefore more information is needed on how granules respond to a wide range of pharmaceuticals.

The pharmaceuticals diclofenac (DCF), erythromycin (ERY), and gemfibrozil (GEM) were selected for use in this study because, though each is frequently detected in the environment (Fang et al., 2012; Schafhauser et al., 2018; Sathishkumar et al., 2020), few studies exist on each compound’s interaction with AGS. Diclofenac is a commonly used non-steroidal anti-inflammatory drug (NSAID) that is poorly removed (under 40%) in conventional wastewater treatment systems (Paxéus, 2004). It has also been shown to act synergistically with antibiotics to prevent biofilm formation (Pawar et al., 2019). Erythromycin is a macrolide antibiotic commonly used in both human and veterinary medicine, and has been shown to bioaccumulate in multiple aquatic species (Schafhauser et al., 2018). Lastly, gemfibrozil is a fibrate, or lipid regulator, that has been shown to inhibit growth and cause endocrine disruption in various aquatic organisms (Zurita et al., 2007). All three compounds have been detected in wastewater treatment plant influents at concentrations as high as 64 μg/L (Fang et al., 2012; Schafhauser et al., 2018; Sathishkumar et al., 2020).

There were three objectives of this study: (1) Identify how the three common, but relatively unstudied, pharmaceuticals listed above impact conventional wastewater treatment by lab-grown AGS; (2) investigate the fate of each pharmaceutical by tracking aqueous and solid phase parent compounds and degradation products; and (3) track microbial community and activity changes throughout exposure. To our knowledge, no studies have confirmed pharmaceutical biodegradation by AGS using detection of degradation products. Notably, the byproducts formed during degradation of pharmaceuticals may be more toxic than the parent pharmaceuticals, and therefore it is vital to improve understanding of the intermediates and products formed. Abiotic removal of the dosed pharmaceuticals was monitored in this study but is not discussed in order to allow sufficient discussion of biotic processes here. In particular, this study sought to link shifts in the active microbial community in pharmaceutical-exposed granules with changes in wastewater treatment efficacy and pharmaceutical fate.

## 2 Methods

### 2.1 AGS reactor operation

AGS sequencing batch reactor (SBR) operation is detailed in Bodle et al., 2022. In brief, AGS was grown in two identical glass SBRs with a working volume of 3.4 L. Both SBRs were operated in repeating three-hour cycles: 72 minutes anaerobic feed, 100 minutes aeration at a gas flow rate of 5 L/min, three minutes settling, and five minutes effluent discharge. The hydraulic residence time in both reactors was approximately 6.4 hours, and the solids residence time was controlled at approximately 25 ± 5 days. SBR operating parameters are consistent with those in other lab-scale studies (e.g., Kong et al., 2015; Margot et al., 2015; Rodriguez-Sanchez et al., 2017). During aeration, pH and dissolved oxygen were controlled with LabVIEW software (National Instruments) at 7.0 ± 0.3 and 1.75 ± 0.25 mg/L, respectively. Influent media were identical to those described by de Kreuk and van Loosdrecht, 2004, except that influent sodium acetate was increased to 10.3 mM, resulting in an organic loading rate of 2.5 g C/L*d. Both SBRs were initially seeded with AGS from an AquaNereda^®^ treatment plant in Utrecht, The Netherlands and operated at steady state (i.e., complete nitrogen and phosphate removal) for over 300 days prior to beginning experimentation. Immediately before starting experimentation, both SBRs were emptied and AGS was combined, mixed, and redistributed so that granule qualities would be as similar as possible in both reactors.

For 80 days, the test reactor received 46 mL of pharmaceutical media with the influent medium, resulting in an influent concentration of approximately 150 μg/L of each pharmaceutical. Individual stock solutions of each pharmaceutical were prepared first in methanol at 10 g/L, then diluted into nanopure water to obtain two pharmaceutical media: one solution (“DG”) containing 17.86 mg/L each of diclofenac sodium (Acros Organics) and gemfibrozil (Acros Organics), and a second solution (“ERY”) containing 35.8 mg/L erythromycin (TCI Chemicals). Twenty-three mL of each solution (46 mL total) were delivered with the influent medium throughout the anaerobic feed period. Approximately 0.13 mg/L methanol was also present in the combined influent stream. The pharmaceutical media were protected from light to prevent photolytic degradation and prepared fresh every 8–10 days.

Results discussed in Bodle et al., 2022 showed that the pharmaceuticals tested herein sorbed to different lab materials with different affinities; therefore, to ensure the accuracy of pharmaceutical dosing, ERY solution was pumped into the reactor using silicone tubing (Masterflex). DG solution was pumped into the reactor using PharmaPure tubing (Masterflex). Despite different pharmaceutical stock solution concentrations, sorption to tubing resulted in fairly stable influent concentrations of approximately 150 μg/L of each pharmaceutical. Although each pharmaceutical is typically measured in wastewater treatment plant influents at approximately 1–10 μg/L (Fang et al., 2012; Schafhauser et al., 2018; Sathishkumar et al., 2020), pharmaceutical sorption to tubing drove usage of this elevated influent concentration: 150 μg/L was the minimum concentration of pharmaceuticals that could be consistently dosed to the test reactor without significant losses from sorption. Influent samples were taken from a sampling port in the tubing located at the base of the reactor (reactor schematic available in Supplementary Information Figure S1) and were extracted and quantified per methods detailed in Section 2.3.

### 2.2 Analytical methods – conventional wastewater analytes

Influent and effluent samples from both reactors were regularly taken and filtered through 0.45 μm regenerated cellulose filters prior to analyses for ammonia, nitrite, nitrate, phosphate, and dissolved organic carbon. Ammonia was quantified with Hach kit TNT 832 and a Hach DR 3900 spectrophotometer, equivalent to US EPA Method 350.1. Other anions listed were quantified with a Dionex ICS-1100 anion chromatography system equipped with an IonPac AS22 RFIC column (4 × 250 mm) and IonPac guard column (4 × 50 mm). Dissolved organic carbon (DOC), defined as that which could pass through a 0.45 μm filter, was measured with a SKALAR Formacs^HT^ Total Organic Carbon analyzer system.

### 2.3 Analytical methods – pharmaceutical analyses

Aqueous influent and effluent samples were prepared for mass spectrometry (MS) analyses by solid phase extraction (SPE) as detailed in Bodle et al., 2022. It is important to note that the periodic flow conditions that are characteristic of SBR systems allow constant sorption and desorption of pharmaceuticals within influent tubing. For this reason, the most accurate method of quantifying influent concentrations would have required extraction of the entire influent volume, which would been too destructive to reactor operation. For that reason, 100 mL influent or effluent sample were filtered with a 1.5 μm glass fiber filter (Hach) to remove solids and loaded on preconditioned Waters Oasis HLB cartridges (30 mg, 20 mL) at 10 mL/min using a vacuum manifold system. Loaded cartridges were washed, dried, and frozen at −18°C until elution (no longer than 14 days). Pharmaceutical losses to the lab materials used during extraction (glassware, syringes, pipette tips, and glass fiber filters) were tested for and were found to be minimal (Bodle et al., 2022).

Influent and effluent pharmaceutical samples were periodically taken in duplicate to account for possible pharmaceutical losses during the extraction process, as detailed in Bodle et al., 2022. In brief, one sample from each duplicate set was pre-spiked with pharmaceutical stock solution to a final nominal concentration of 100 μg/L prior to extraction. After extraction, the unspiked sample was split in half, and one half was post-spiked to a final nominal concentration equal to 100 μg/L multiplied by each samples’ concentration factor. Recovery was then determined as follows:

$$\text{Recovery}=\frac{\text{Prespike concentration}-\text{unspiked concentration}}{\text{Postspike concentration}-\text{unspiked concentration}}$$

Measured pharmaceutical concentrations in unspiked samples were thereby corrected for recovery of each analyte. Recovery was 97 ± 9% for DCF, 117 ± 17% for ERY, and 98 ± 11% for GEM (Table 1). Triplicate influent and effluent samples were taken once per month to assess accuracy. Pharmaceutical quantification and detection were performed with an Acquity I Class Plus ultra-performance liquid chromatograph (UPLC) coupled to a Waters Synapt XS quadrupole time-of-flight mass spectrometer (QToF-MS) in positive ion mode. Chromatographic analysis methods were adapted from Sodré and Sampaio, 2020. In brief, pharmaceuticals were separated with an Agilent Eclipse Plus C18 column (2.1 × 100 mm) at 30°C under gradient elution. Formic acid-enriched methanol and ultrapure water (0.1% v/v) were used as mobile phases at 0.7 mL/min, and the concentration of organic solvent was increased from 1% initially to 95% at 10.1 minutes, then returned to initial conditions and re-equilibrated for 3 minutes (total run length of 13 minutes). Sample injection volume was 8 μL. Retention times are summarized in Table 1.

A calibration curve relating each pharmaceuticals’ peak area with its nominal concentration in analytical standards was used to determine parent compounds’ concentrations. Standards were prepared in both methanol and water. The instrument limits of quantification were under 10 μg/L for all compounds. The open-source software MZmine and R were used to analyze and compile mass spectra data (RStudioTeam, 2009; Pluskal et al., 2010).

All samples were also screened for DCF, ERY, and GEM biodegradation products using a personal compound database and library (PCDL) developed from the literature (SI Table S1). Degradation products are reported when mass errors were less than 5 ppm and the signal-to-noise ratio of peaks was greater than or equal to 10. The goal of this approach is not to quantify degradation products’ concentrations but simply to use the presence of degradation products as an indicator of biodegradation. The relative concentrations of aqueous degradation products were tracked over time by calculating corrected peak areas as follows:

$$\text{Aqueous corrected peak area}=\frac{\text{Degradation product peak area}\times \text{Concentration factor}}{\text{Peak area of relevant }100\;\frac{\mu g}{\text{L}}\text{ standard}}$$

where the “relevant standard” term refers to the peak area of the related parent compound at 100 μg/L. For example, the peak area of an aqueous ERY degradation product would be divided by the peak area of the 100 μg/L ERY standard measured during the same mass spectrometry run and multiplied by the sample’s concentration factor (e.g., a 100 mL sample extracted and concentrated to 1 mL had a concentration factor of 100).

Pharmaceuticals were also extracted from granules to quantify solid phase concentrations per methods adapted from Martin et al., 2010. Extraction methods are summarized in the Supplementary Information. All solid phase samples were screened for degradation products as described above. Solid phase samples were extracted in triplicate once per month and periodically evaluated for recovery using spike-recovery testing, as described above. Average recoveries are summarized in Table 1. Peak areas of degradation products in the solid phase were corrected as follows:

$$\text{Solid corrected peak area}=\frac{\text{Degradation product peak area}}{\text{Peak area of relevant }100\frac{\mu g}{L}\text{ standard}\times \text{Sample dry weight }(g)}$$

Aqueous and solid samples were periodically analyzed from the control reactor for pharmaceutical parents and degradation products. Except when noted otherwise, pharmaceuticals were not detected in any phase in the control reactor. Likewise, degradation products were generally not found in the influent to the test reactor.

Lastly, the toxicities of detected degradation products were estimated using the US Environmental Protection Agency’s Toxicity Estimation Software Tool (TEST). TEST estimates chemical toxicity using quantitative structure activity relationships (Martin et al., 2020).

### 2.4 Bacterial community composition

#### 2.4.1 DNA/RNA extraction

Granules from both reactors were periodically sampled for molecular characterization of the prokaryotic microbial community using high throughput sequencing of 16S rRNA genes and transcripts. Granules were collected during the aeration phase to ensure samples were fully mixed and representative of communities in the entire reactor. Samples were stored at −80°C prior to extraction. Nucleic acids were extracted from approximately 10 granules at each time point. Extraction and analyses of replicate nucleic acid samples were beyond the scope of this study. All samples were extracted at once by bead beating in DNA/RNA Shield (Zymo Research), then DNA was purified from the lysate with the DNA Clean and Concentrator Kit (Zymo Research). RNA samples were extracted from the same lysate with the Direct-zol RNA Miniprep Kit (Zymo Research), digested with the TURBO DNA-free Kit (Invitrogen), and purified with the RNA Clean and Concentrator Kit (Zymo Research). RNA was then reverse transcribed to cDNA using the ProtoScript II First Strand cDNA Synthesis Kit (New England Biolabs). DNA and RNA concentrations were quantified with Qubit dsDNA HS and RNA HS kits (Invitrogen), respectively. For all kits listed, the manufacturer-provided protocols were followed.

#### 2.4.2 Metagenome characterization

Metagenomic sequencing of granules was conducted on granular inoculum approximately 300 days before beginning the experimentation described herein; therefore, metagenome data provides an approximation of microbe functionality within granules (given the time between metagenome sampling and the onset of the experiment). DNA was extracted using the Zymobiomics DNA Miniprep kit and quantitated with the Qubit dsDNA kit (Invitrogen). Libraries were prepared for shotgun metagenomic sequencing on the Illumina NovaSeq platform using paired end 150bp sequencing at Novogene with a target of 15Gb of data. To quality filter fastq files, prinseq was used to remove sequences with more than 10 Ns, mean quality scores below 20, sequences shorter than 50 nt, and to trim ambiguous bases from the ends of reads. Paired end sequences were then assembled using metaSPades (v 3.15.5, Nurk et al., 2017) with kmer sizes from 21 to 99 and all reads were mapped against the resulting scaffolds using Bowtie2 (Langmead and Salzberg, 2012). The sam files were converted to bam format using samtools (Li et al., 2009). We binned scaffolds into putative metagenome assembled genomes (MAGs) using MetaBat2 (v 2.2.15, Kang et al., 2019). CheckM (v 1.2.2, Parks et al., 2015) was used to calculate MAG quality, the relative abundance of each bin (based on the number of reads mapped to each MAG), and to construct a multigene phylogeny. Any MAG with completeness greater than 50% and with contamination above 10% was refined using Anvio (v 7.1, Eren et al., 2015). To classify the taxonomy of putative bins, we used GTDB-tk (Chaumeil et al., 2019) against the v207 database release.

To gain insights into the potential functional pathways contained within the abundant MAGs, putative genes were identified using prodigal and compared to the Kyoto Encyclopedia of Genes and Genomes (KEGG) with the program KOFAM scan (Aramaki et al., 2019). Pathway completeness measures were generated using KEGG Decoder (Graham et al., 2018). This analysis was focused on MAGs with completeness over 90%, combined with MAGs that had lower completeness but made up over 1% of the community. Our analysis focused primarily on nitrogen cycling, based on previous findings showing shifts in these pathways in reactors with pharmaceutical-exposed wastewater (Jiang et al., 2021).

#### 2.4.3 16S library preparation and sequencing

To characterize the prokaryotic community, Phusion Hot Start II DNA polymerase (Thermo Scientific) was used to target the V4 region of the 16S rRNA gene with the primers 515F-A (GTGYCA GCMGCCGCGGTAA) and 806R-b (GGACTACVSGGGTATC TAAT). Reactions were 20 μL each with final concentrations of 0.4 mM dNTPs, 0.2 mM primers, and 1 U polymerase. Thermocycling conditions consisted of an initial denaturation at 98°C for 10 seconds followed by 30 cycles of denaturing at 98°C for 20 seconds, annealing at 60°C for 10 seconds, and elongating at 72° C for 30 seconds. A final extension was then performed at 72°C for 5 minutes. PCR products were purified with Mag-Bind TotalPure NGS beads (Omega), and samples were then barcoded with the Nextera XT Index Kit v2 Set D (Illumina). Barcoded samples were again purified with Mag-Bind beads, quantified, and pooled at equimolar concentration into a sample library containing approximately 30 ng DNA from each sample. Sequencing was performed onsite at Montana State University with the Illumina MiSeq platform using the v3 600 cycle kit. Raw sequence files were deposited to GenBank (BioProject ID PRJNA985155).

#### 2.4.4 Statistics and data analysis

USEARCH software was used to merge forward and reverse reads, quality filter with a max score of 1, trim primer sequences, and dereplicate sequences. The UNOISE3 algorithm was used to identify zero-radius OTUs (ZOTUs) and construct an OTU table. To classify ZOTUs, we used SINTAX against a reference database of 16S sequences from the Genome Taxonomy Database (GTDB, release 202, Parks et al., 2021) with outgroup sequences for chloroplast and mitochondria. Sequences identified as Eukarya or with a bootstrap value less than 70% at the phylum level were removed from downstream analyses. A phylogenetic tree was constructed using a reference maximum likelihood tree generated from full length and near-full length 16S sequences downloaded from GenBank and GTDB using RAxML (Stamatakis, 2014). MAFFT (Katoh and Standley, 2013) was used to align the OTUs to the reference sequences; OTUs were then inserted into the reference tree with pplacer (Matsen et al., 2010).

The goal of targeting both rRNA genes and transcripts (cDNA) was to examine both the total microbial community, defined as the community recovered in DNA reads, and the active microbial community, defined as ZOTUs with a ratio of 16S rRNA transcripts to rRNA genes greater than or equal to 1 (Bowsher et al., 2019). Rarefied ZOTU matrices of rRNA transcripts (cDNA) and rRNA genes paired by sample were used to calculate the transcript to gene ratios and identify active OTUs across 100 rarefaction trials. To account for so-called phantom taxa, or taxa detected in rRNA but not rRNA gene sequences, values were set to 100 based on methods described in Bowsher et al., 2019 prior to calculating mean values across trials. Due to the potential for bias in sequence numbers arising during cDNA transcription, DNA read numbers were used to calculate diversity indices. DNA read numbers for phantom taxa were also included in the active community. To examine differences among the community pools, non-metric multidimensional scaling ordination was used to compare the total (DNA-based), RNA-based, and active communities. Taxa were grouped at the family level to calculate changes in relative abundance over time.

To link shifts in the active microbiome with changes in nitrogen and phosphorus levels, as well as pharmaceutical degradation, we used vector fitting of effluent concentrations of DCF, ERY, and GEM using the function envfit in vegan (Oksanen, 2010). More specifically, to examine if specific microbial groups could be linked to the strong decline in pharmaceutical removal observed between days 5 and 17, we calculated response ratios of the active ZOTUs with greater than 0.1% relative abundance between these time points. These values ranged from −1 to 1, with 1 being a strong increase in relative abundance at day 17, and −1 being a strong decrease. Values between −0.5 and 0.5 were considered neutral. The calculated values were added to the phylogeny as a dataset within the ITOL annotation platform to examine the potential for cohesive negative or positive responses within specific clades. All statistical analyses were conducted using R software (version 4.2.2, RStudioTeam, 2009) with packages vegan (Oksanen, 2010), picante (Kembel et al., 2010), and phyloseq (McMurdie and Holmes, 2013).

## 3 Results

### 3.1 Pharmaceutical impacts on granular wastewater treatment performance

**FIGURE 1:**
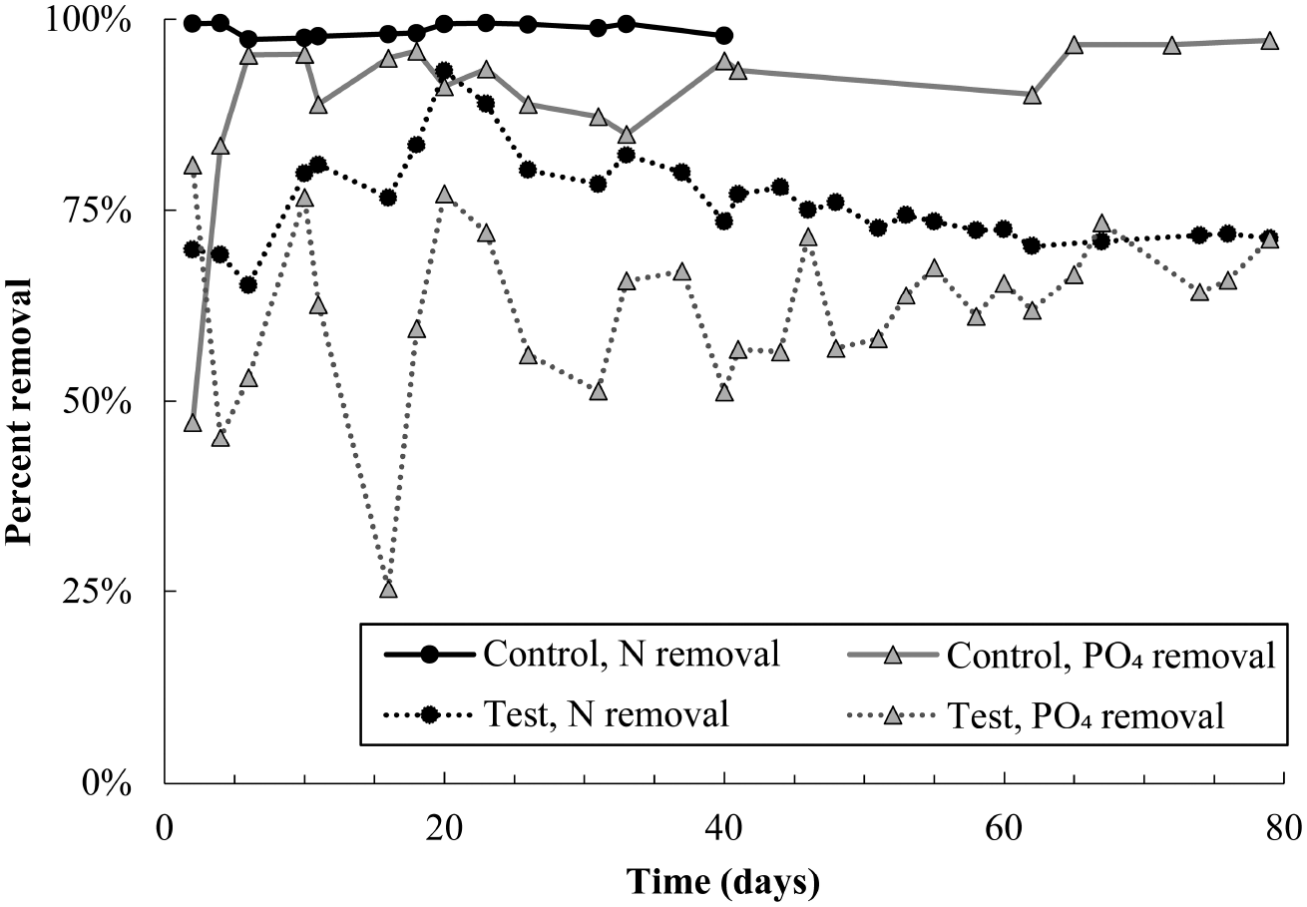
Total nitrogen and phosphate removal in the control and test SBR during the pharmaceutical dosing period. Note that N removal data in the control reactor is not plotted after day 40 because an acid overdose in the control reactor severely inhibited nitrifying populations. Given trends prior to the acid overdose, it is likely that nitrogen removal in the control would have proceeded at 100% if not for this issue. Nitrogen and phosphate removal in the test reactor averaged out at 73 ± 2% and 63 ± 6%, respectively, over the last 40 days of the experiment.

Immediately after pharmaceutical dosing to the test reactor began, nitrogen removal dropped to approximately 70% (Figure 1). There was a brief recovery in nitrogen removal from day 6 to 20, during which removal peaked at 93%, though removal declined thereafter and stabilized at approximately 70%. Poor nitrogen removal was due mainly to incomplete ammonia oxidation (SI Figure S2). Nitrite and nitrate concentrations in both control and test reactors were approximately equal for the entire experimental duration (Figure S3), indicating that nitrite oxidation and denitrification were minimally impacted by pharmaceutical exposure. Nitrogen removal data for the control reactor are not plotted past day 40 because the software controlling both reactors experienced an error on day 40, stopping dissolved oxygen control in the test reactor and resulting in an acid overdose to the control reactor. DO control to the test reactor was interrupted for just 24 hours; however, the acid overdose to the control reactor caused shutdown of that reactor for 10 days. It is reasonable to expect that complete nitrogen removal would have continued in the control reactor if not for this disruption.

Phosphate removal also declined sharply in the test reactor and remained noisy for the entire experimental duration (Figure 1). Despite this, DOC consumption was over 90% in both reactors (Figure S4), and most carbon continued to be consumed anaerobically in the test reactor (Figure S5). Near-complete anaerobic carbon consumption suggests the potential activity of glycogen accumulating organisms, discussed more in section 4.1.

### 3.2 Pharmaceutical removal

**FIGURE 2:**
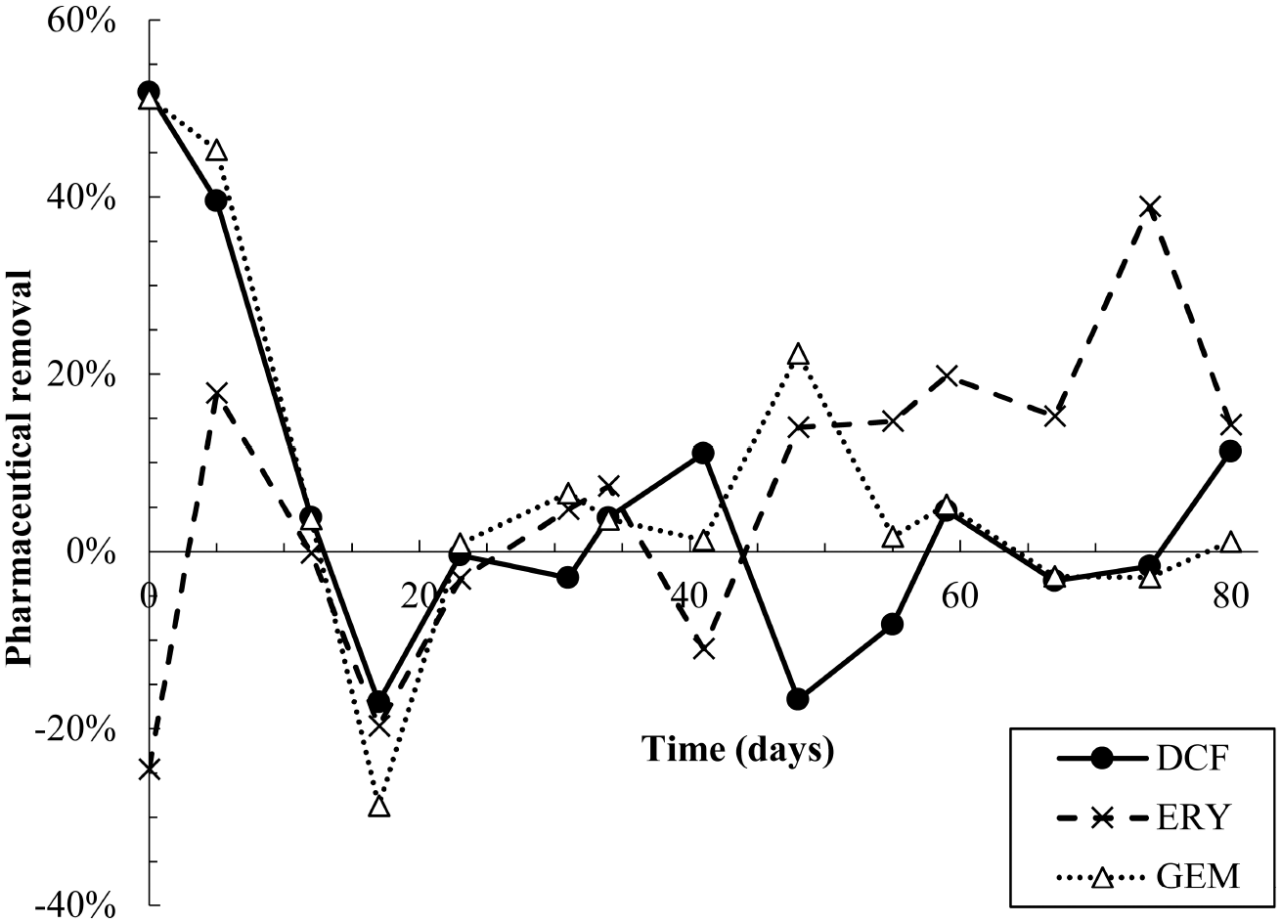
Pharmaceutical removal versus time. Removal was calculated based on a mass balance using measured influent and effluent concentrations (SI equations S1 and S2). Negative removal percentages indicate that effluent concentrations were higher than predicted by the mass balance.

**FIGURE 3:**
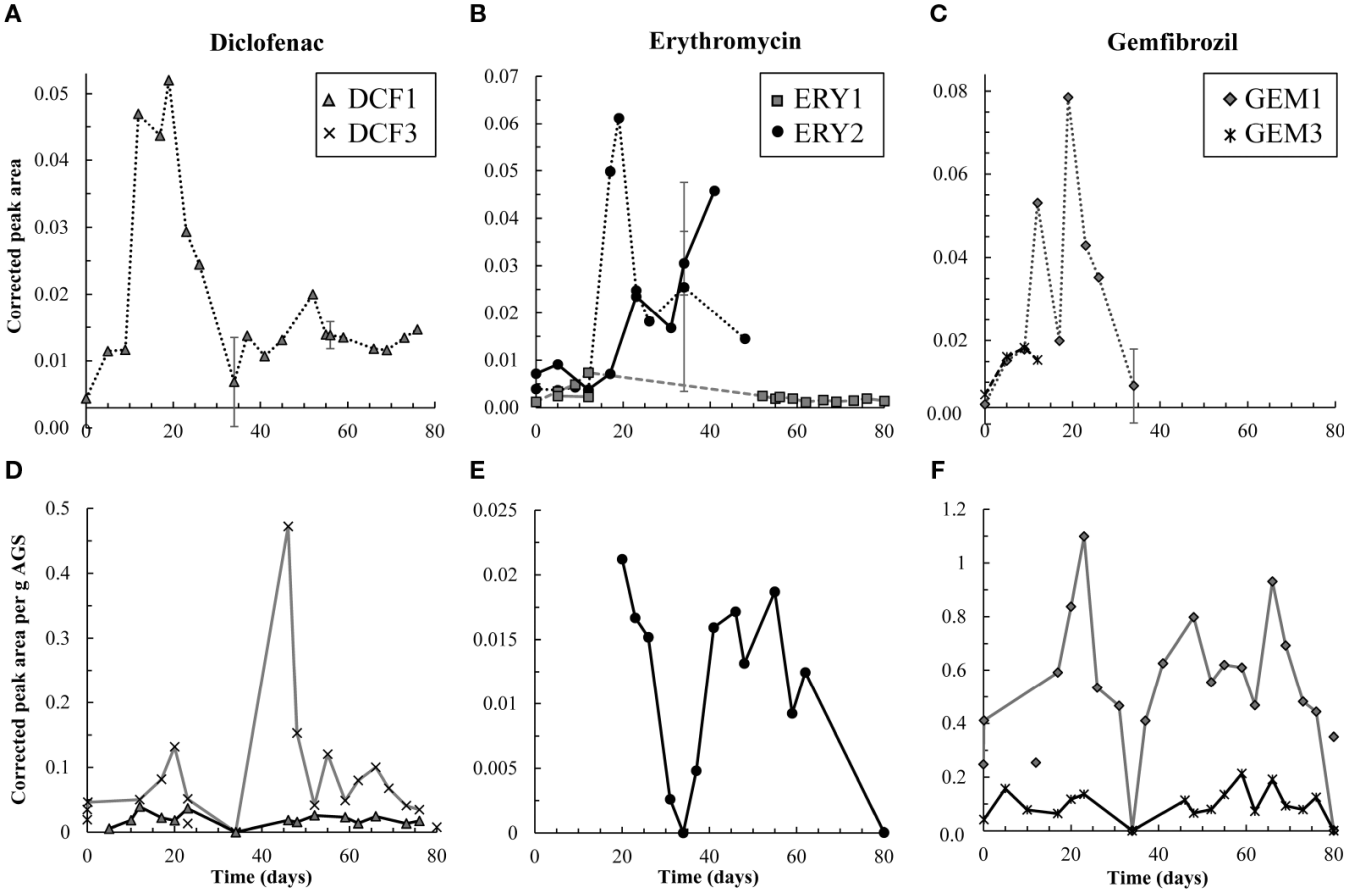
Top row, **(A-C)** aqueous degradation products detected over time in the effluent (dashed and dotted lines) from the test reactor for DCF, ERY, and GEM, respectively. Y-axes all reflect corrected peak area but differ in scale. ERY-associated degradation products were temporarily detected in the influent (solid lines) to the test reactor. However, corrected peak areas of both ERY-associated products were generally higher in the effluent than influent, and therefore AGS-driven biodegradation of these compounds was likely occurring. Bottom row, **(D-F)** solid phase degradation products for DCF, ERY, and GEM, respectively. Note that y-axes differ in scale though each reflects corrected peak area per g AGS. Points not connected by a line indicate detections in control granules. DCF3 was detected three times in control granules, likely due to cross-contamination during mass spectrometry analyses. Likewise, GEM1 was detected twice in control granules. Error bars, representing standard deviation of triplicate samples, are present for both aqueous and solid data on days 34, 56, and 80, and at times are smaller than sample points. Points on these days are averages.

All pharmaceuticals were partially removed in the first 10–12 days of dosing, evidenced by lower effluent than influent concentrations (Figure 2, Figure S6). Removal was calculated by performing a mass balance on influent and effluent concentrations at each time point (SI Equations S1 and S2). Degradation products were also detected during the first 12 days (Figure 3). However, from day 12–23, effluent concentrations of all pharmaceuticals spiked to approximately twice influent concentrations, indicating a release of retained pharmaceuticals from the reactor. Solid phase data collected over this time frame also appear to indicate desorption of pharmaceuticals from AGS (Figure S6). Pharmaceutical degradation is discussed in more detail in the following sections.

#### 3.2.1 Diclofenac

Pharmaceutical fate was most clearly interpreted for DCF: over the first 12 days, solid phase DCF concentrations increased, while effluent concentrations remained lower than influent ones, and removal peaked at approximately 50% (Figure 2). The DCF degradation products hydroxy-DCF (C_14_H_11_Cl_2_NO_3_, “DCF1”) and 1-(4-hydroxyphenyl)-2-,3-dihydro-1H-indol-2-one (C_14_H_11_NO_2_, “DCF3”) were also present in the aqueous and solid phases in the first 12 days (Figure 3). Taken together, these data indicate removal via sorption and biodegradation.

From days 17–23, desorption and a decline in biodegradation capacity likely caused DCF to spike in the effluent: on days 17 and 19, effluent concentrations peaked while solid phase DCF concentrations sharply declined (Figure S6), suggesting desorbing DCF contributed to higher concentrations in the effluent. Likewise, aqueous phase DCF1 peaked from days 12–19 (Figure 3), suggesting that bacteria in AGS were less capable of converting this product to further intermediates. Solid phase peak areas of DCF1 also increased slightly over the same timeframe, likely because aqueous phase concentrations were higher and therefore increased sorption was possible.

Influent and effluent DCF concentrations were near equal from day 23 on, indicating negligible DCF removal. Interestingly, aqueous and solid phase DCF degradation products were detected for the remainder of the experiment. The presence of these products may indicate partial biodegradation, albeit not at rates significant enough to result in measurable removal.

The degradation products detected represent initial and tertiary products, according to a pathway proposed in Jewell et al., 2016: DCF1 is formed first via mono-oxygenation. DCF1 may be present as two isomers, 4’-hydroxy-DCF and/or 5-hydroxy-DCF, but the UPLC-QToF-MS method used herein was unable to differentiate between the two. It is most likely that DCF1 was present as the 4’ isomer, as DCF3 is formed from degradation of this isomer. 4’-hydroxy-DCF is then converted to 1-(2-chloro-4-hydroxyphenyl)-3H-indol-2-one (C_14_H_10_NO_2_Cl, “DCF2”) via reductive dechlorination and amidation, and DCF3 is then formed via further reductive dechlorination (Jewell et al., 2016). Further degradation of DCF3 is hypothesized in Jewell et al., 2016, but intermediate structures are unknown.

Pharmaceutical degradation products may have had an inhibitory effect on bacteria in AGS; however, the impacts of many degradation products on wastewater bacteria or other environmental receptors are not well understood. Regarding the herein-detected DCF degradation products, a study by Syed et al., 2016 showed that 4’-hydroxy-DCF inhibits ATP synthesis in rat liver mitochondria, though concentrations tested were higher than would be expected in humans and significantly higher than those in the environment. Conversely, 4’-hydroxy-DCF did not have any inhibitory impact on *Vibrio fischeri* bacteria at up to 20 mg/L (Grandclément et al., 2020). The toxicity of DCF3 has not been established in the literature, therefore TEST was used. Toxicity estimates for all detected degradation products are summarized in SI Table S2. In general, the predicted toxicities for detected degradation products are similar to or slightly less toxic than those for DCF, with the exception of DCF1, which is predicted to be more bioaccumulative and toxic to fathead minnows.

#### 3.2.2 Erythromycin

Similar to DCF, ERY was removed in the first 12 days of dosing via both sorption and biodegradation. Effluent concentrations were lower than influent ones for the first five days, and aqueous phase degradation products were also measured at low levels over the initial 12-day period (Figure 2, Figure 3, Figure S6). Notably, ERY removal was negative on day zero, indicating that measured effluent concentrations were higher than those predicted by a mass balance (Figure 2). It is probable that negative removal on day zero is an artifact of noisy influent pharmaceutical concentrations, as discussed in Section 2.3 and shown in Figure S6. Influent ERY concentrations on day zero were likely higher than measured, given that influent concentrations then stabilize at approximately 207 ± 22 μg/L for the next 20 days. Between days 12–23, aqueous ERY degradation products then spiked, as did effluent ERY concentrations, evidenced by negative removal (Figure 3).

Low levels of the primary and secondary products 3-depyranosyloxy ERY (C_29_H_53_NO_10_, “ERY1”) and 7,12-dyhydroxy-6-deoxyerythronolide B (C_21_H_38_O_8_, “ERY2”), respectively (Llorca et al., 2015; Ren et al., 2021), were present up to day 12, indicative of biodegradation. On days 17 and 19, ERY2 concentrations spiked in the effluent, likely indicating that conversion of this secondary product to further intermediates was no longer occurring at the same capacity. Interestingly, both products were also present in the influent (solid lines in Figure 3B). Influent ERY1 concentrations were lower than effluent ones, indicating that AGS contributed to formation of ERY1. ERY2 concentrations were higher in the influent than effluent and continued to increase until day 40. The presence of both ERY1 and ERY2 in the influent likely indicates photodegradation or ERY biodegradation by contaminating biomass in the influent tubing. ERY2 was also present in the solid phase throughout the test.

After day 20, effluent ERY concentrations declined to near influent ones. Approximately 19 ± 9.7% removal was sustained after day 48, perhaps due to slightly elevated solid phase concentrations over the same time (Figure 2, Figure S6). The presence of aqueous and solid phase ERY degradation products for the remaining test duration may also indicate partial removal via biodegradation.

ERY degradation pathways potentially used by wastewater bacteria and proposed in Ren et al., 2021 and Ren et al., 2022 hypothesize that ERY1 is formed first via cleavage of the cladinose sugar from ERY. Cladinose was not detected in this study, likely because it is readily metabolizable. ERY1 then undergoes further degradation to ERY2 via cleavage of the desosamine sugar from ERY1, and the final product of ERY degradation is 2,4,6,8,10,12-hexamethyl-3,5,6,11,12,13-hexahydroxy-9-ketopentadecanoic acid (C_21_H_40_O_9_, “ERY3”). Desosamine and ERY3 were also not detected, again likely because both are readily metabolizable, or because the mass spectrometry method used was not suitable for these compounds. Although ERY removal peaked at 18% in the first 12 days of dosing, the low levels of both ERY1 and ERY2 detected in that period suggest that intermediates completed the degradation pathway and end products were fully metabolized.

The TEST-predicted toxicities of ERY1 and ERY2 are summarized in SI Table S2. ERY1 and ERY2 appear to be less toxic to aquatic organisms than ERY, though both are predicted to be more toxic to rats.

#### 3.2.3 Gemfibrozil

Much like DCF and ERY, GEM removal occurred due to a combination of sorption and biodegradation in the first 12 days of dosing, followed by a spike in effluent concentrations from days 12–23 (Figure 2). Removal was then near zero for the remainder of the test. Aqueous GEM degradation products were not detected in the effluent after day 34 (Figure 3). The primary and tertiary products 5-[2-(hydroxymethyl)phenoxy]-2,2-dimethylpentanoic acid (C_15_H_22_O_4_, “GEM1”) and 2-[(4-carboxy-4-methylpentyl)oxy] benzoic acid (C_15_H_20_O_5_, “GEM3”), respectively (Kjeldal et al., 2016), were both measured up to day 12. GEM1 then spiked in the effluent. This pattern also mimics that seen for DCF and ERY degradation products: Primary products were converted to downstream intermediates until day 12, after which conversion stopped occurring to the same extent, causing primary products to wash out in the effluent.

The same primary and tertiary products were present in the solid phase for the entire experimental duration (Figure 3), which may indicate a preference of these compounds for the solid phase and/or continuous low levels of biodegradation. Regardless, biodegradation was not significant enough to impact GEM removal. GEM1 was also detected twice in control granules, likely due to carry over during mass spectrometry analyses: GEM1 was not detected in control influent or effluent, and relative solid phase concentrations were lower in control samples than test granules.

Kjeldal et al., 2016 isolated a GEM-degrading *Bacillus* species from activated sludge and proposed a degradation pathway in which GEM is first converted to GEM1 via hydroxylation. GEM1 is next oxidized to 5-(2-formylphenoxy)-2,2-dimethylpentanoic acid (GEM2), which is then further oxidized to GEM3. According to the degradation pathway proposed in Kjeldal et al., 2016, GEM3 degradation may undergo two to three further reactions before reaching final products. Based on this, the GEM degradation observed in the first 12 days may have proceeded to approximately 50% completion, though degradation rates were insufficient for complete removal. Further GEM degradation may have also occurred, but UPLC-MS methods may not have been suited to detect other products. TEST-predicted toxicities of GEM2 and GEM3 are summarized in SI Table S2. Predicted toxicities and bioconcentration factors are generally lower for degradation products than for GEM.

### 3.3 Functional potential of granules from shotgun metagenomics

Metagenomic sequencing generated approximately 25 million high quality reads, resulting in a total of 19 bins with greater than 90% completeness and less than 5% contamination, and an additional 38 with completeness greater than 50% with less than 10% contamination. All MAGs were classified as members of the bacteria, including known members of glycogen accumulating organisms (GAOs) such as species in the *Competibacter* and *Contendibacter*. Phosphorus accumulating organisms (PAOs) were also present, such as species within the *Accumulibacter*. A MAG identified as a species in the *Accumulibacter* was the most abundant organism detected, at 19% of the population based on the number of raw reads that mapped to the MAG.

**FIGURE 4:**
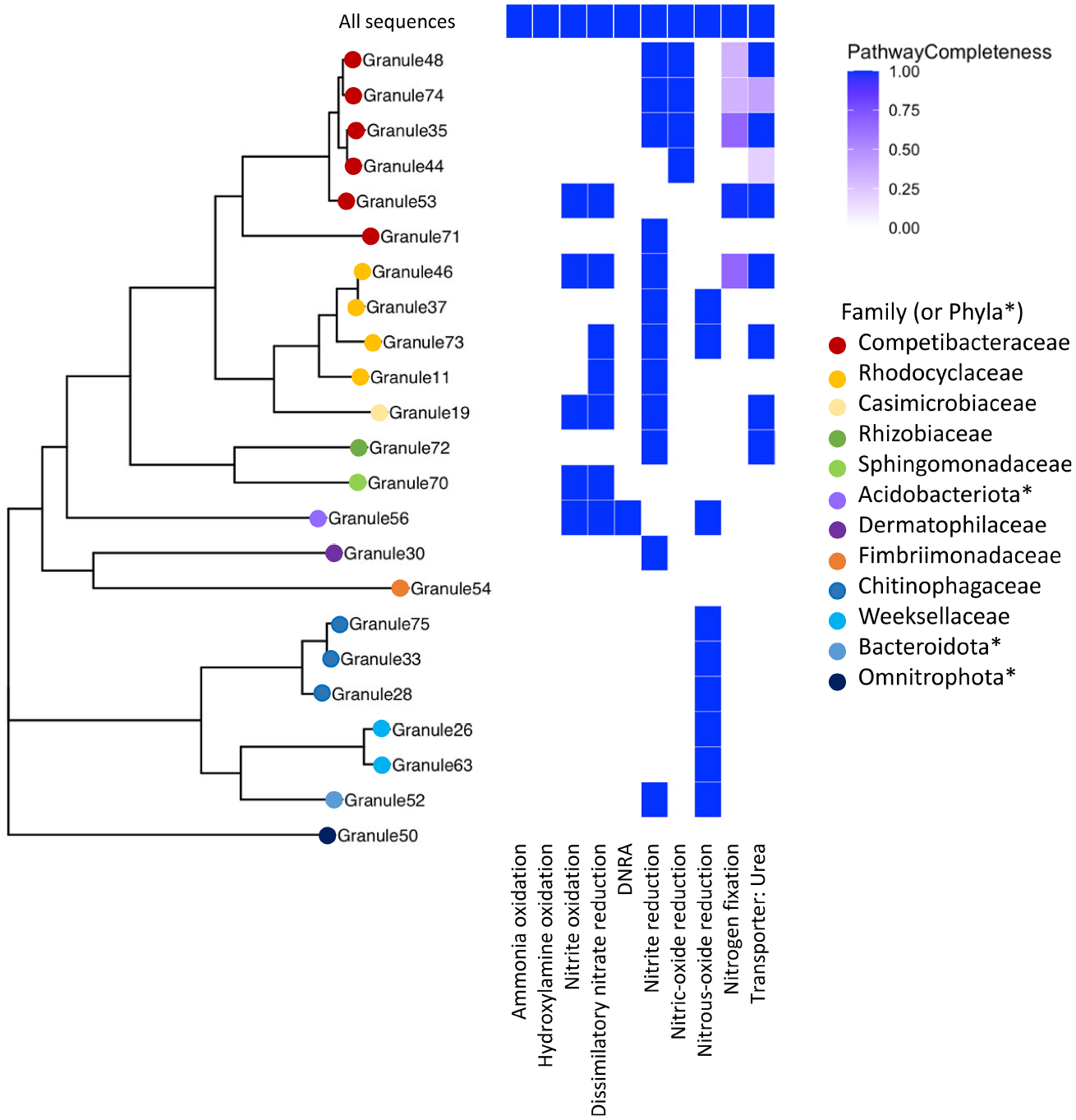
A multigene phylogeny of assembled MAGs and the level of completeness for nitrogen cycling pathways. Organisms identified from the assembled MAGs were from a range of families, with a large number in the Pseudomonadota (e.g., Competibacteraceae, Rhodocyclaceae) and the Bacteroidota. *Phyla, rather than family, are listed when the GTDB-provided family name corresponds to an uncultivated organism. None of the assembled MAGs contained the ammonia oxidation pathway, but multiple MAGs had complete denitrification pathways, primarily for dissimilatory nitrate reduction. Note that granule numbering is arbitrary and was simply used to differentiate different samples.

Despite the presence of multiple MAGs identified as known ammonia oxidizing bacteria (AOB), including *Nitrosospira* and *Nitrosomonas*, this pathway was not uncovered in the KEGG analysis of constructed MAGs, though the complete ammonia oxidation pathway was found in the total shotgun community (Figure 4). The scaffolds with identified ammonia monooxygenase (*amo*) genes (*amoA, amoB*, and *amoC*) were relatively short (< 6000 bp). One scaffold contained all three *amo* genes, and another scaffold contained just *amoA* and *amoC*. Based on BLAST (Altschul et al., 1990), both were highly similar to sequences identified from *Nitrosomonas* species; one scaffold was similar to a MAG identified as *Nitrosomonas* from a metagenomic analysis of biofilms in wastewater treatment plants (Suarez et al., 2022). The absence of this pathway in putative AOB in the assembled MAGs could be due to the relatively low genome completeness calculated for these taxa, as the *Nitrosomonas* MAG (Granule57, Figure 4) was 87% complete; however, the relative abundance of this organism was 0.66%, and was not included in the overall KEGG analysis. In addition, this MAG had the pathway for nitrite reduction and nitric oxide reduction associated with NOB. We also identified the complete pathway for hydroxylamine oxidation, the second step in nitrification, in the total community, but not in the assembled MAGs.

Nitrate reductase genes were widespread in the granule community, as seen in previous metagenomic studies of wastewater (Singleton et al., 2021). Twelve MAGs contained genes for the complete dissimilatory nitrate reduction pathway and of those, ten also contained the full nitrite oxidation pathway (Figure 4) including members of the *Rhodocyclaceae*, a *Competibacter*, and a MAG classified as a member of the Thermoanaerobaculia. Only a single MAG (Granule56) had the pathway for dissimilatory nitrate reduction to ammonia (DNRA), identified as a species in the Thermoanaerobaculia in the phylum Acidobacteriota, which also contained the dissimilatory nitrate reduction pathway. An organism related to the Thermoanaerobaculia was also detected in the active community, but at very low levels (< 0.1% relative abundance).

### 3.4 Active microbial community response to pharmaceuticals

Shifts in the active microbial community were evaluated using 16S sequencing of rRNA genes and transcripts throughout pharmaceutical exposure. As described in Section 2.4.4, the active microbial community was defined as ZOTUs with a ratio of 16S rRNA transcripts (i.e. cDNA) to rRNA genes greater than or equal to one. The 16S rRNA gene read numbers from ZOTUs identified as “active” were then used to calculate relative abundances within the total active community. Multidimensional ordination (SI Figure S7) showed separation of the three pools (DNA, RNA, and active), but also showed separation of the treatment and control SBRs.

**FIGURE 5:**
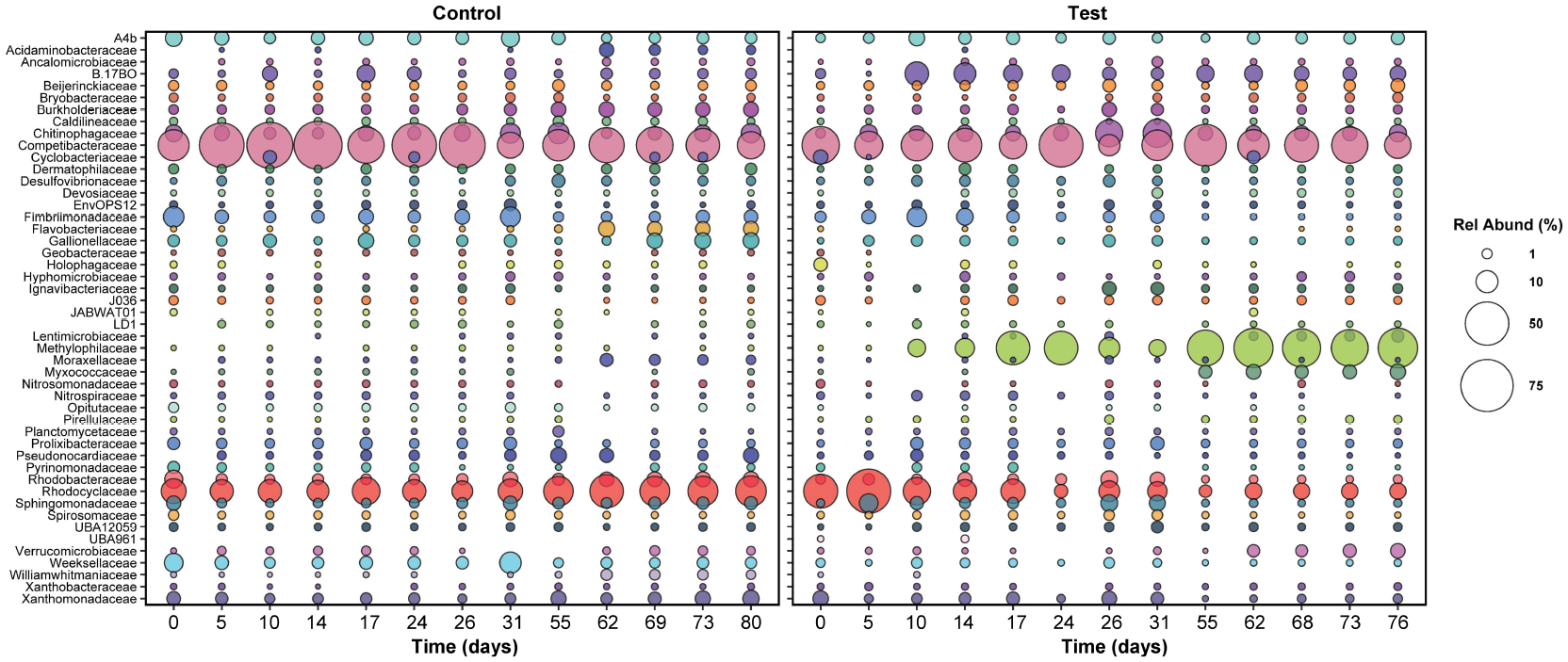
The relative abundance of active families in both reactors over time. Only families with relative abundance greater than or equal to 1% at at least one time point are plotted. Sampling times were matched across both reactors; for this reason, relative abundance data between days 31 and 55 are not plotted, since the control reactor was shut down during this time.

At day zero, the distributions of active families in both the control and test reactors were similar: *Competibacteraceae*, *Rhodocyclaceae*, and *Chitinophagaceae* were most abundant (Figure 5). Notably, the *Rhodocyclaceae* family includes PAOs (*Candidatus Accumulibacter)* and a potential denitrifying genus (*Zoogloea*). The role of bacteria in the *Chitinophagaceae* family is unclear—multiple OTUs matching *Niabella*, *Terrimonas*, and *Flavipsychrobacter* genera were detected, but to date, the function of these genera in AGS is unknown.

After five days, active families in the test and control SBRs diverged greatly. In the test reactor, active *Rhodocyclaceae* spiked in abundance at day five, from 14 to 46% (Figure 5), which may be linked with the brief recovery in phosphate removal between days four and 10 (Figure 1). Active *Rhodocyclaceae* then declined sharply in abundance in the test reactor for the remainder of the dosing period, stabilizing at approximately 1.5 ± 0.7% abundance for the second half of the test. In contrast, active *Rhodocyclaceae* in the control reactor were present at 10.2 ± 5% for the entire test duration.

Despite large shifts in the test reactor community, significant correlations between microbial community composition and effluent pharmaceutical concentrations were generally not found. A significant, though small, correlation between effluent GEM concentrations and the microbial community was observed (R^2 =^ 0.28, p = 0.048).

Despite the lack of overall correlations, when times with the largest differences in pharmaceutical removal (specifically days 5 through 17, Figure 2) were focused on, the strongest declines in relative abundance were concentrated within the Gammaproteobacteria and primarily in *Rhodocyclaceae* (Figure 5, Figure S8). The most striking shift for any single ZOTU was observed for a sequence classified as a member of *Azonexus* (family *Rhodocyclaceae*), which made up 40% of the active community at day 5 but was absent by day 17. Large changes in relative abundance were also observed for two members of *Accumulibacter*, which were present at over 1% of the active community on day 5 but were not detected by day 17.

In contrast, positive responses were distributed across the phylogeny, with the largest shift occurring in a ZOTU identified as a *Methylotenera* species: relative abundance increased from under 0.1% at day 5 to 22% at day 17. Likewise, a ZOTU identified as a Bacteroidia species (UBA2475_sp013816615) increased from under 0.1% to 6% over the same time period. Positive shifts were also observed for members of the Chloroflexota, Gammaproteobacteria, and Acidobacteriota (Figure S8).

We had limited success linking the ZOTUs to the organisms identified in the metagenome assemblies. Of the top ten most abundant ZOTUs in the active community, three had no close relatives in the metagenome. The most abundant active ZOTU in the test reactor (genus *Methylotenera*, order Burkholderiales), was not detected in the assembled MAGs; however, this is likely due to the length of time elapsed between metagenome sampling and the onset of experimentation as well as the presence of pharmaceuticals. Four ZOTUs were classified as *Competibacter A denitrificans* with varying levels of confidence. The remaining ZOTUs with a close relative in the metagenome included a *Xanthomonadaceae* (genus SCMT01), a species of *Accumulibacter*, and an *Azonexus* species.

## 4 Discussion

### 4.1 Links between active bacterial community, wastewater treatment, and pharmaceutical degradation

*Methylophilaceae* became the dominant active family in the test reactor after day 17 and included *Methylotenera* and *Methylophilus* genera. Members of this family may have proliferated due to the presence of trace methanol in the influent from pharmaceutical dosing (0.13 mg/L influent concentration); however, some *Methylophilaceae* species can also consume acetate (Jenkins et al., 1987; Dedysh et al., 2005), and therefore it is more likely that *Methylophilaceae* proliferated due to increased acetate availability in the aerobic phase of SBR operation (Figure S5). Some *Methylotenera* species are also capable of aerobic denitrification (Mustakhimov et al., 2013), which may explain why nitrate did not accumulate in the test reactor. It is worth noting that this family did not appear to be affected by pharmaceuticals’ presence, but also did not proliferate until after pharmaceutical removal stopped—*Methylophilaceae* were therefore likely not responsible for the pharmaceutical degradation observed in the first 12 days of the test.

*Competibacteraceae* was the second most abundant active family in the test reactor, at 21.8 ± 10.5% abundance over the last 40 days of exposure. Bacteria in the *Competibacteraceae* family are glycogen accumulating organisms, or GAOs, that compete with PAOs for anaerobic carbon consumption but do not aerobically consume phosphate. For this reason, their proliferation is typically linked with reduced phosphate removal (McIlroy et al., 2014). It is likely that *Competibacteraceae* activity sustained anaerobic carbon consumption in the test reactor; however, it is surprising that the relative abundance of this family was lower in the test reactor than in the control reactor: active *Competibacteraceae* were present at 30.8 ± 18.6% abundance in the control reactor, despite complete phosphate removal (Figure 1). It is possible that *Competibacteraceae* activity levels were similar in both reactors; the increased abundance of active *Methylophilaceae* in the test reactor may make the relative abundance of active *Competibacteraceae* appear smaller.

**FIGURE 6:**
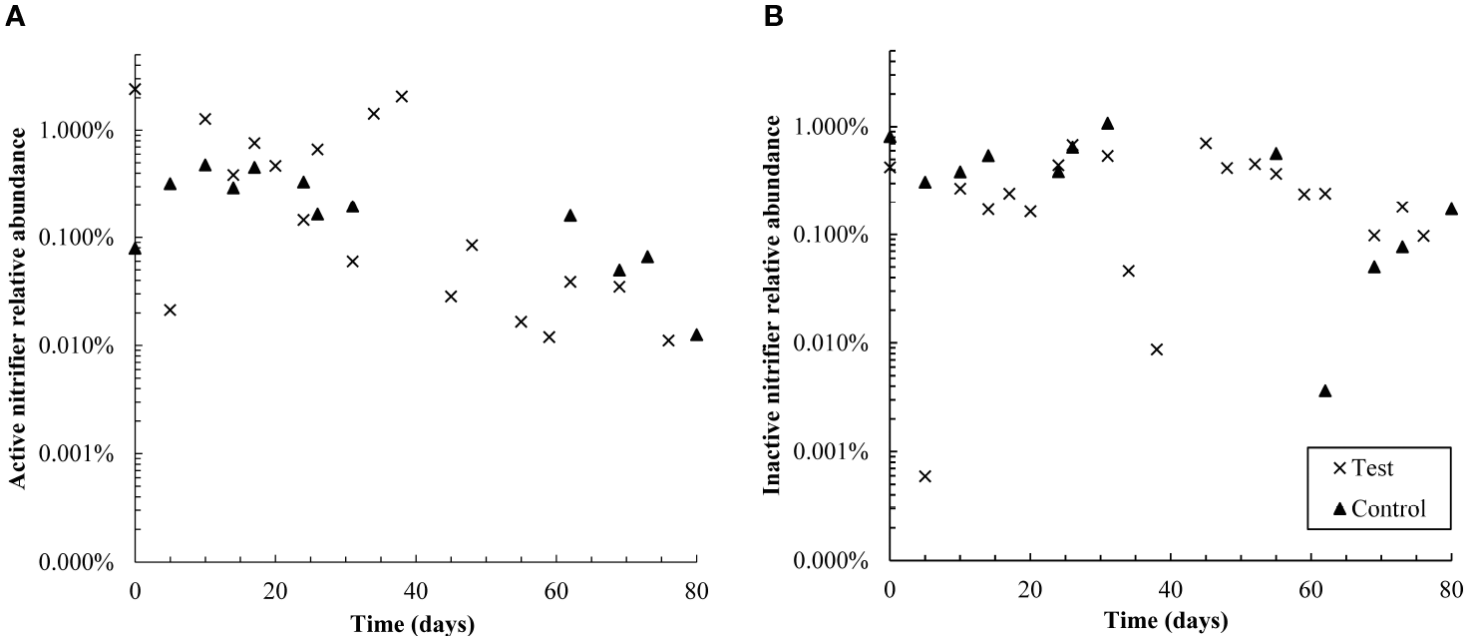
Semi-log plots of the relative abundance of active **(A)** and non-active **(B)** nitrifiers (summed Nitrosomonadaceae and Nitrospiraceae families) over time in control and test reactors. Over the first 38 days, active nitrifiers were more abundant in the test reactor than the control; however, from days 55–80, active nitrifiers were more abundant in the control SBR. Active nitrifiers also declined in relative abundance in the test reactor throughout the dosing period. The control SBR was shut down from days 40–50 and therefore data is not available in that timeframe; this is also likely why active nitrifiers in the control reactor are present at slightly lower relative abundances in the second half of the experiment than the first. Inactive nitrifiers were present at similar levels in both reactors throughout the test.

The relative abundance of active nitrifying families *Nitrospiraceae* and *Nitrosomonadacea* was higher in the test reactor than the control for the first 38 days of pharmaceutical exposure (Figure 6), which may explain why nitrogen removal briefly recovered between days zero and 20 (Figure 1). Nitrifier activity may also be responsible for pharmaceutical degradation in the first 12 days of the test: the ammonia monooxygenase enzyme used by both ammonia oxidizing bacteria and archaea (AOA) is known to react non-specifically with aromatic compounds, typically through an oxidation reaction (Keener and Arp, 1994; Schatteman et al., 2022). The primary degradation product of DCF, and all detected GEM degradation products, are formed through oxidation reactions. Aqueous GEM degradation products were not detected after day 34, approximately the same time that active nitrifier abundance dropped. Likewise, aqueous DCF1 levels fell and remained low after day 34. It is possible that decreased levels of DCF and GEM degradation products after day 34 are linked with decreased abundance of active nitrifiers over the same time period. It should be noted, however, that active *Nitrosomonadaceae* (the only AOB family detected) were not detected for most timepoints in both the control and test SBR. The lack of active AOB data, however, is contradicted by complete ammonia oxidation in the control reactor and partial ammonia oxidation in the test reactor, as well as active *Nitrospiraceae* abundance patterns over the dosing period. Several active AOA genera were also detected, which may further explain why ammonia oxidation continued throughout dosing. AOA abundance is discussed in more detail in section 4.2.

### 4.2 Patterns in taxonomic shifts in microbial communities

The addition of pharmaceutical compounds led to strong shifts in the microbial community and concomitant reductions in nitrogen and phosphorus removal. Based on the assessment of potential functional pathways, no high-quality MAGs were found with the ammonia oxidation pathway. Although AOB, such as *Nitrosomonas*, were detected in both the shotgun metagenomic and metabarcoding datasets, we were unable to construct a MAG with over 90% completeness, which limited our ability to determine the role of these organisms. However, despite their limited abundance overall, it is likely that *Nitrosomonas* is the main driver of ammonia oxidation in the reactors, given their presence in seed granules and the homology of the identified functional genes.

Several archaeal taxa were also found in the metabarcoding dataset though archaea were not detected in the granule metagenome. Archaea detected were primarily species within the *Thermoproteota*, including members of the order *Nitrosophaerales*. *Nitrosophaerales* contains numerous AOA, such as *Nitrosotenuis* and *Nitrosocaldus* genera. AOA have been posited to be important for the oxidation of ammonia to nitrite in commercial wastewater treatment plants (Limpiyakorn et al., 2013), though a study by Bai et al., 2012 found that AOA dominated in smaller scale treatment plants while AOB dominated in commercial ones. The authors hypothesized that AOA may be more sensitive to the toxic compounds more frequently measured in larger scale treatment plants.

Although archaea made up less than 1% of the sequences identified, 47 ZOTUs were identified as active taxa. We focused primarily on abundant taxa, but the ability of rare organisms to drive ecosystem function is well documented (Jousset et al., 2017); for example, rare taxa may be particularly important in pollutant degradation. The large shifts in relative abundance of wastewater-treating taxa, such as *Rhodocyclaceae* and *Methylophilaceae* (Figure 5), suggest that additional studies in which microbe functionality is explored could provide important insights into community interactions that might otherwise be overlooked.

## 5 Conclusions

Lab-grown AGS was exposed to three commonly found, but relatively unstudied pharmaceuticals at approximately 150 μg/L each. The fate of each pharmaceutical and its degradation products in the aqueous and solid phases were monitored, and pharmaceutical impacts on wastewater treatment performance and microbial communities were evaluated.

All pharmaceuticals were partially removed via both biodegradation and sorption in the first 12 days of the study. Biodegradation capacity then declined irreversibly, indicated by washout of degradation products, declining production, and negligible pharmaceutical removal. Exposure to the pharmaceutical mixture negatively impacted wastewater treatment efficacy and the relative abundance of active wastewater treating families. Nitrogen and phosphate removal declined to approximately 73% and 63%, respectively, though carbon removal was not impacted. Declining nitrogen removal was due mainly to inhibited ammonia oxidation and likely also related to the declining abundance of active nitrifiers. Similarly, active *Rhodocyclaceae* declined in abundance in the test reactor, which likely contributed to poor phosphate removal.

## Supplementary Material

Supplementary Material

## Figures and Tables

**TABLE 1:** Physical and chemical properties of tested pharmaceuticals, as well as average extraction recoveries and retention times.

	Diclofenac	Erythromycin	Gemfibrozil
Chemical formula	C_14_H_11_Cl_2_NO_3_	C_37_H_67_NO_13_	C_15_H_22_O_3_
Molecular weight (g/mole)	296.1	733.9	250.3
Octanol-water partition coefficient (Log K_ow_)	4.51 (Avdeef et al., 1998)	3.06 (Schafhauser et al., 2018)	4.77 (Fang et al., 2012)
Aqueous phase extraction recovery	97 ± 9%	117 ± 17%	98 ± 11%
Solid phase extraction recovery	75 ± 8%	56 ± 22%	68 ± 8%
UPLC retention time (minutes)	8.8	6.8	9.5

## Data Availability

The datasets presented in this study can be found in online repositories. The names of the repository/repositories and accession number(s) can be found below: https://www.ncbi.nlm.nih.gov/genbank/, PRJNA985155.
