# Supplementary material for: Treatment performance and microbial community structure in an aerobic granular sludge sequencing batch reactor amended with diclofenac, erythromycin, and gemfibrozil
Source: Front Microbiomes. Author manuscript; Available in PMC 2023 Dec 8. (PMC10705044; doi:10.3389/frmbi.2023.1242895)
Supplement: Supplementary Material [file NIHMS1948503-supplement-Supplementary_Material.pdf]

SUPPLEMENTARY INFORMATION

Treatment performance and microbial community structure in an aerobic granular sludge sequencing batch reactor amended with diclofenac, erythromycin, and gemfibrozil

Authors: Kylie B. Bodle[1,2*], Rebecca C. Mueller[2,3], Madeline R. Pernat[1,2], Catherine M. Kirkland[1,2]

[1]Department of Civil Engineering, 205 Cobleigh Hall, Montana State University, Bozeman, MT, USA

[2]Center for Biofilm Engineering, 366 Barnard Hall, Montana State University, Bozeman, MT, USA

[3]USDA Agricultural Research Service, Western Regional Research Center, Albany, CA, USA

*Corresponding author

Email: kyliebodle@montana.edu

Aerobic granular sludge sequencing batch reactor schematic:

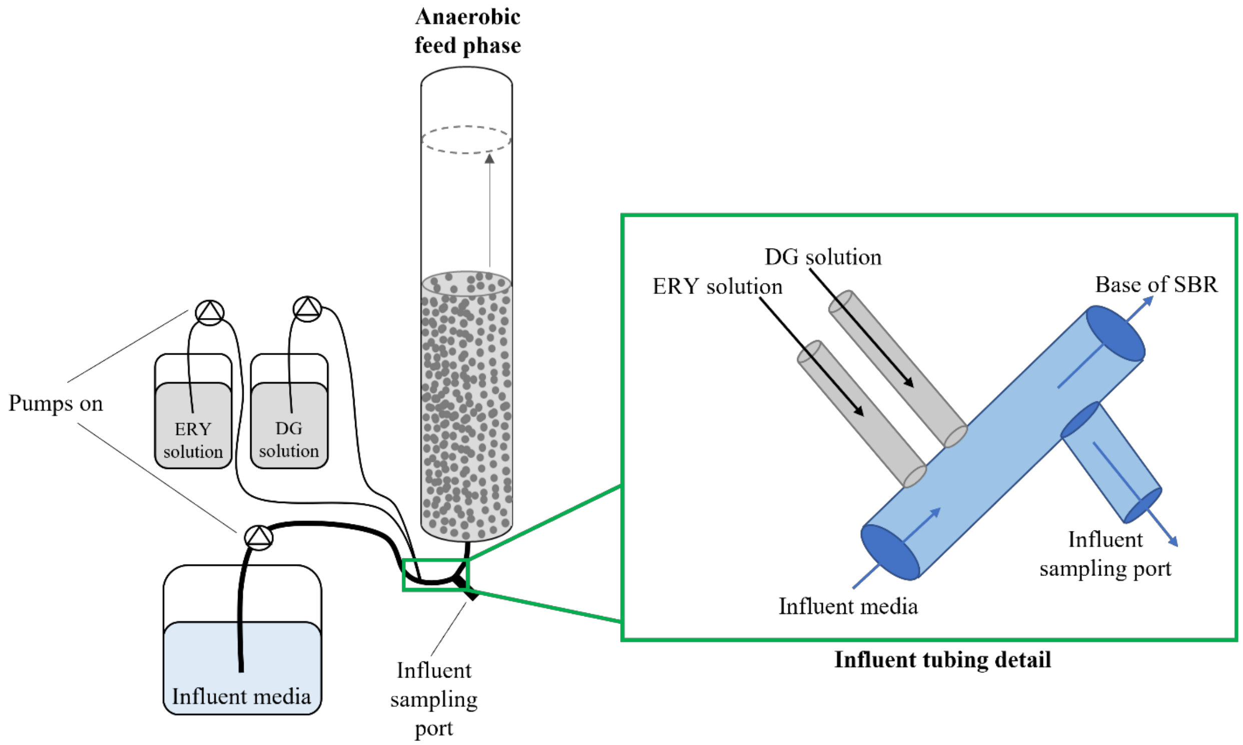

Figure S1. Schematic of the sampling port from which influent samples were taken. During the anaerobic feed phase, the influent sampling port tubing was briefly unclamped to allow collection of samples for influent pharmaceutical and nutrient concentrations.

Suspected diclofenac (DCF), erythromycin (ERY), and gemfibrozil (GEM) degradation products:

Parent compound	Formula	Adduct	m/z	Retention time (min)	Source
ERY	$C_{21}H_{38}O_8$	M+H	419.2639	8.07	[1]
	$C_8H_{16}O_4$		177.1121	-	
	$C_8H_{17}NO_3$		176.1281	-	
	$C_{29}H_{53}NO_{10}$		576.3742	5.5	[1, 2]
	$C_{21}H_{40}O_9$		437.2745	8.7	[1, 3]
	$C_6H_{14}NO$		117.1148	-	[3]
	$C_8H_{16}NO_2$		135.1254	-	
	$C_{29}H_{50}O_{10}$		559.3477	7.1	
DCF	$C_{14}H_{11}Cl_2NO_3$	M+H	312.0189	7.84	[4]
	$C_6H_4Cl_2$		146.9763	-	
	$C_{13}H_9Cl_2NO_2$		282.0083	8.83	[5]
	$C_{14}H_9Cl_2NO_3$		310.0035	7.1	

	$C_{14}H_{11}NO_2$		226.0856	10.07	
	$C_{12}H_9Cl_2NO_3$		286.0031	-	
	$C_{12}H_{11}Cl_2NO_3$		288.0191	-	
	$C_{14}H_{10}NO_2Cl$		260.0467	-	
	$C_{14}H_9Cl_2NO_2$		294.0082	-	
	$C_{13}H_9Cl_2NO_3$		298.0032	-	
	$C_{11}H_9Cl_2NO_3$		274.0032	-	
	$C_{10}H_7Cl_2NO_2$		243.9922	-	
	$C_{14}H_{10}Cl_2NO$		279.0212	8.83	[6]
	$C_{13}H_{10}Cl_2N$		251.0263	8.83	
GEM	$C_{15}H_{22}O_4$	M+H	267.1591	8.6	[7]
	$C_{15}H_{21}O_4$		266.1513	7.78	
	$C_{15}H_{20}O_5$		281.1384	8.83	
	$C_8H_8O_3$		153.0546	-	
	$C_7H_8O_2$		125.0597	-	
	$C_7H_{12}O_4$		161.0808	-	
	$C_6H_6O_4$		143.0339	9.52	
	C_5HO_3		109.9998	-	
	$C_{15}H_{22}O_4$	M+Na	289.1413	8	
	$C_{15}H_{21}O_4$		288.1335	-	
	$C_{15}H_{20}O_5$		303.1206	8.5	
	$C_8H_8O_3$		175.0368	-	
	$C_7H_8O_2$		147.0419	11.64	
	$C_7H_{12}O_4$		183.063	-	
	$C_6H_6O_4$		165.0161	5.1	
	C_5HO_3		131.982	11.65	

Table S1. Suspected degradation products screened for in all samples. This PCDL was constructed after reviewing relevant literature. Not all compounds listed were detected (indicated by "-"). Compounds with the same residence time could not be distinguished from one another and therefore were not reported. Both sodium and hydrogen adducts of gemfibrozil degradation products were often detected; for samples in which both adducts were present, peak areas were summed together and this total area was corrected as described in the main article.

Supplementary methods – pharmaceutical extraction from granules:

Pharmaceuticals were extracted from granules to quantify solid phase concentrations per methods adapted from [8]. In brief, granule samples were split in half, weighed, and crushed. Moisture fractions in half of the sample were determined by drying. The remaining wet half of the sample was sonicated in 5 mL methanol in a glass centrifuge tube for 15 minutes, then centrifuged at 1600 xg for 8 minutes. Supernatant was collected and transferred to a clean glass test tube. Sonication and centrifugation were then repeated with 2 mL methanol followed by 2 ml acetone. Supernatants were collected and combined after each centrifugation, then evaporated down to approximately 2 mL under a gentle nitrogen stream at 40°C. The remaining supernatant was then diluted with 150 mL nanopure water and extracted as described in the main text, section 2.3.

Removal calculation for all analytes (pharmaceuticals and nutrients):

$$Theoretical\ effluent\ concentration = \frac{(Influent \times 1.6L) + (Effluent \times 1.8L)}{3.4\ L} \quad (S1)$$

$$\%\ Removal = \left(1 - \frac{Effluent}{Effluent_{theoretical}}\right) \times 100\% \quad (S2)$$

Results:

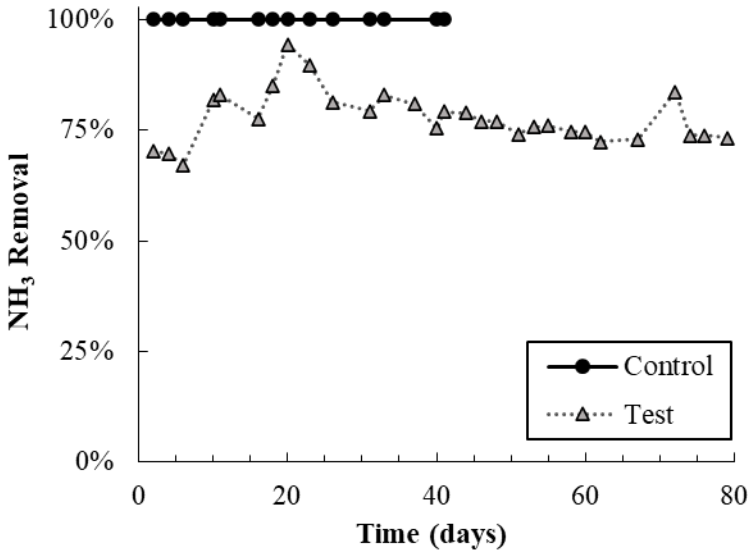

Figure S2. Ammonia removal throughout the dosing period in the control and test reactor. Ammonia concentrations in the control reactor are not reported after day 40 due to a reactor operation issue, discussed in the main article body. Ammonia oxidation in the test reactor was the most inhibited nitrogen removal process—nitrite oxidation and denitrification proceeded at levels near equal to those in the control reactor, evidenced by equivalent nitrite and nitrate concentrations in both reactors (Figure S3).

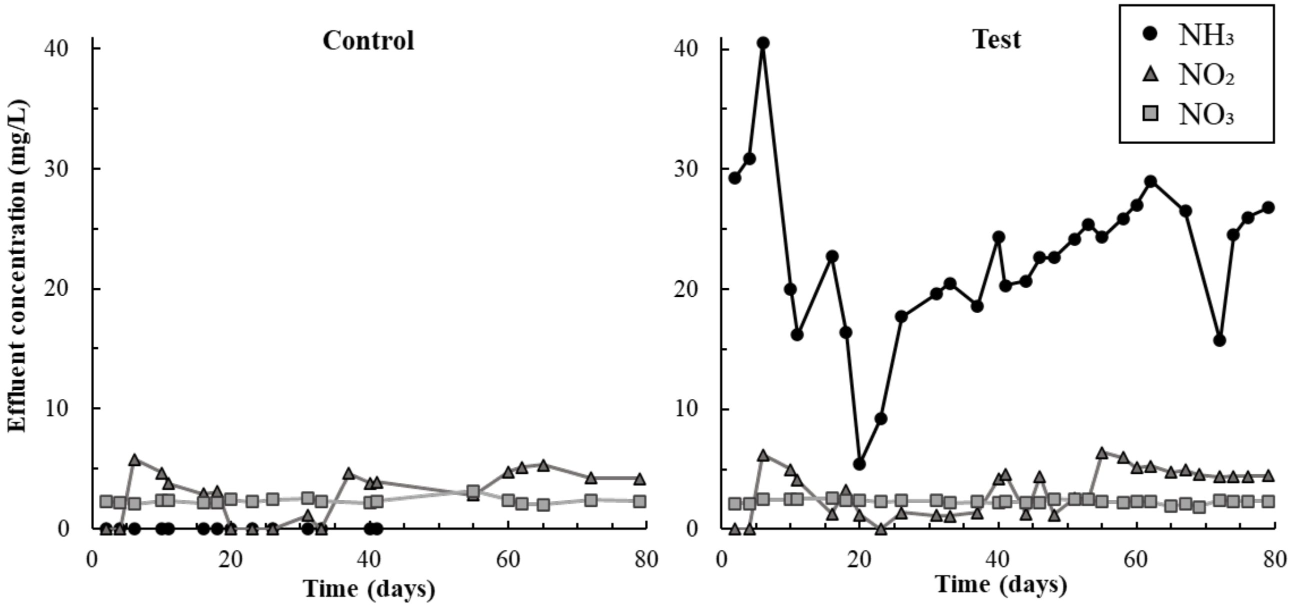

Figure S3. Effluent concentrations of ammonia (NH_3), nitrite (NO_2), and nitrate (NO_3) versus time in the control and test reactors. Effluent ammonia concentrations are not reported past day 40 in the control reactor for reasons discussed previously (see Figure S2 caption).

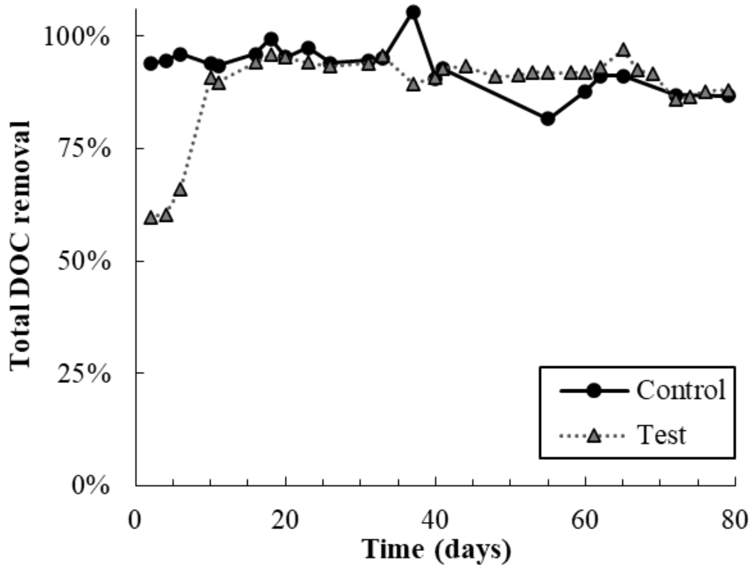

Figure S4. Total dissolved organic carbon (DOC) removal throughout the dosing period in both SBRs. Note that excess DOC was accidently fed in the first 10 days, resulting in lower DOC removal by the test reactor.

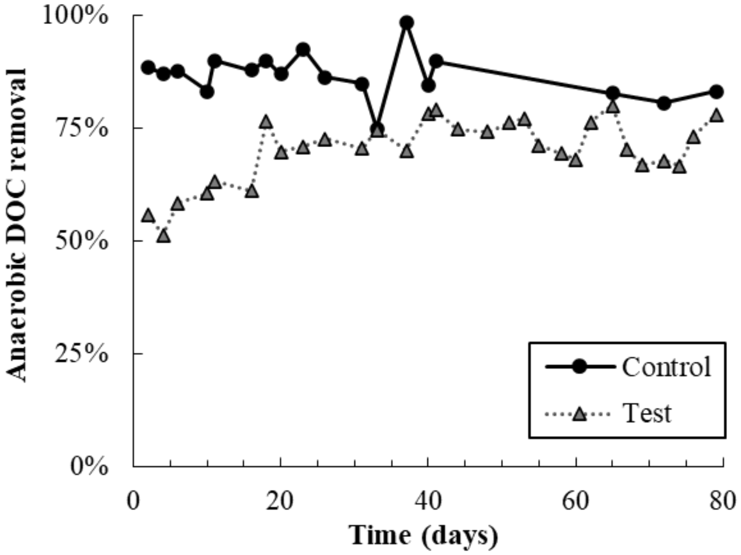

Figure S5. DOC consumption in the anaerobic feed phase of reactor operation. Though more DOC was consumed anaerobically in the control reactor than the test, most DOC consumption in the test SBR continued to occur anaerobically (70 ± 7%).

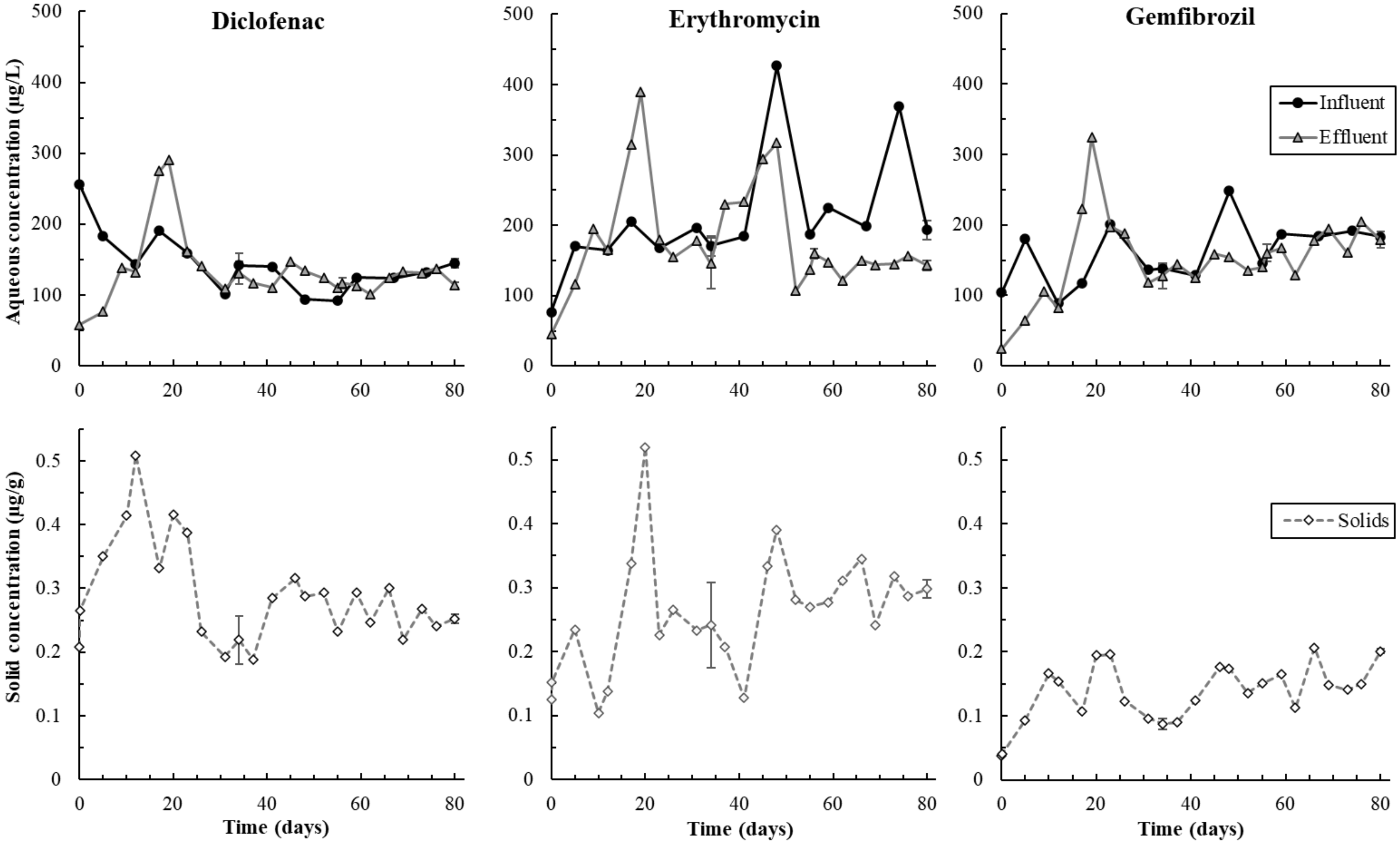

Figure S6. Parent pharmaceutical concentrations in the aqueous (top row) and solid phase (bottom row) over time. Error bars represent the standard deviation of triplicate samples and are present on days 34, 56, and 80; points on these days are averages.

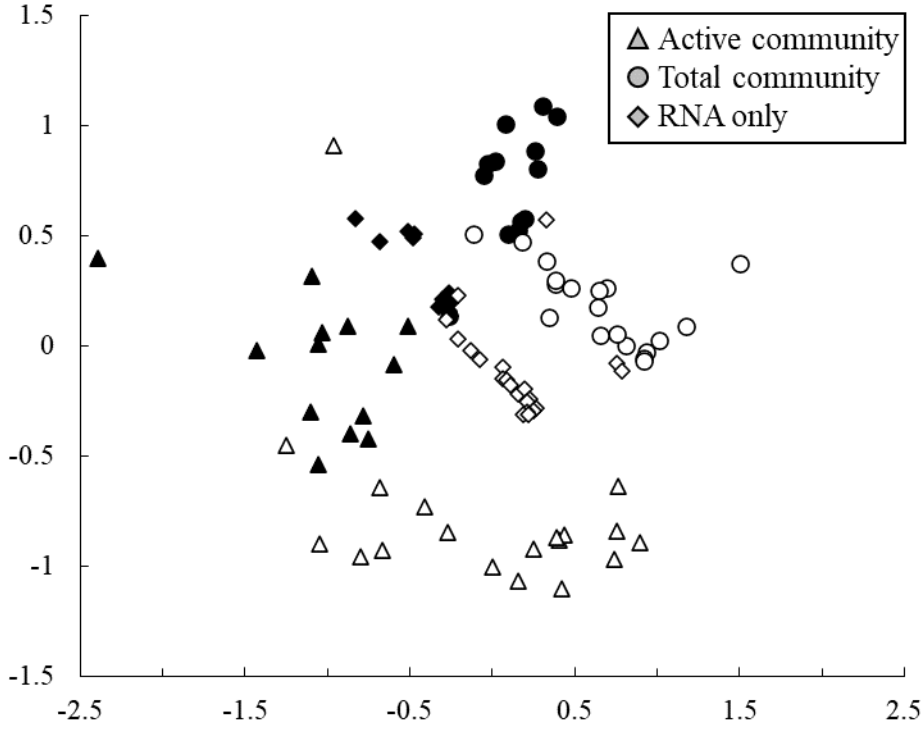

Figure S7. Non-metric multidimensional ordination of community similarity for the control (closed symbol) and test (open symbol) communities. The active community is delineated based on rRNA transcript to gene ratios (triangle symbols); the total community on DNA reads (circles); and lastly the total RNA pool is shown with diamond symbols.

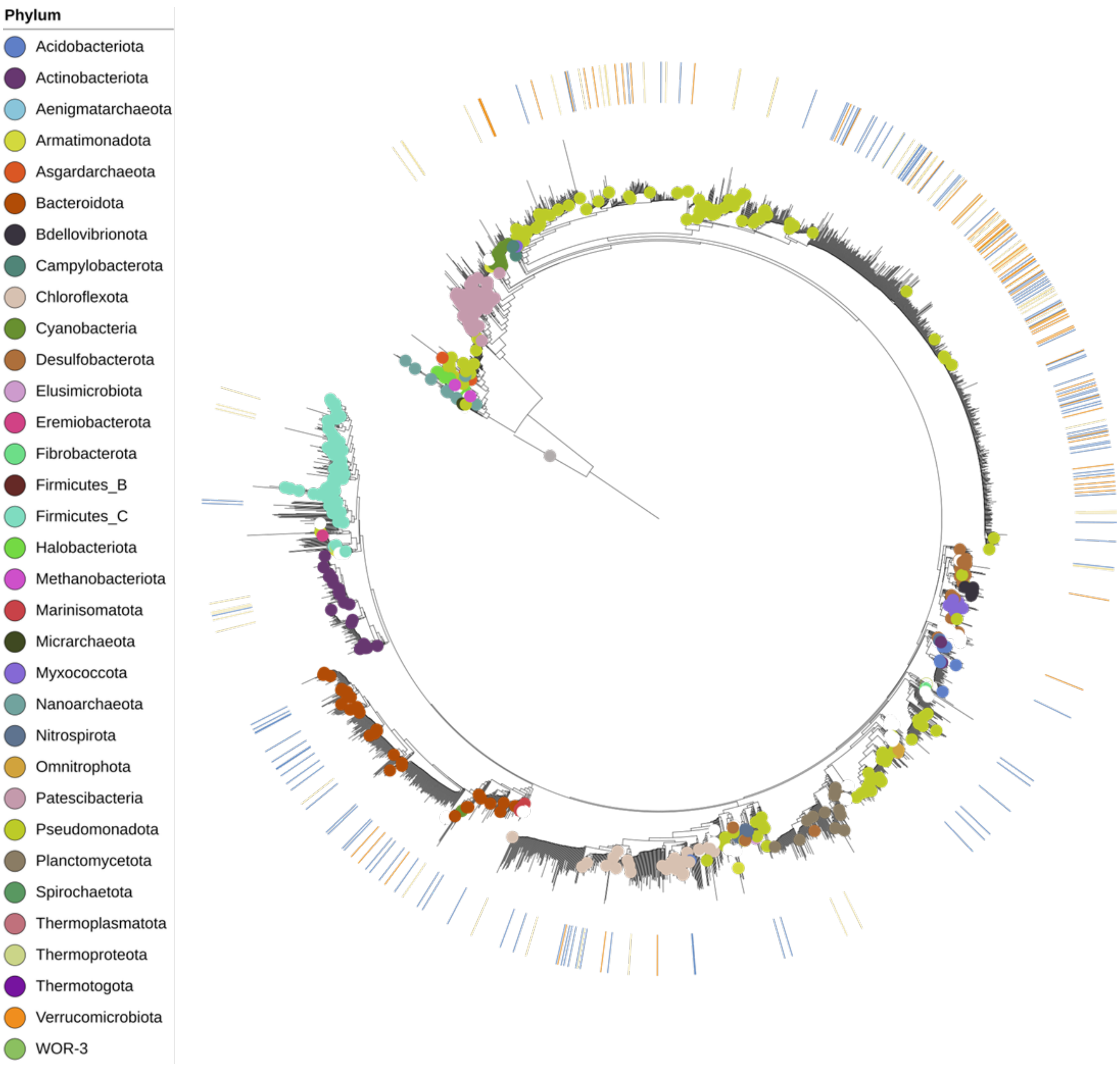

Figure S8. Phylogenetic tree of reference 16S sequences downloaded from GTDB (v 202) with environmental ZOTUs inserted into the tree. Reference taxa are identified with colored symbols. The ring active taxa show negative (orange), neutral (beige), or positive (blue) responses between sampling day 5 and 17, when removal of all pharmaceuticals showed a sharp decline.

Table S2. Predicted toxicity of detected degradation products using EPA TEST software

Colors indicate prediction confidence - key provided below table				Values predicted with EPA TEST software					Mean Absolute Error (MAE) values				
**Name**	**Formula**	**Structure**	**SMILES**	**Fathead minnow LC50 (96 hr, mg/L)**	**Daphnia magna LC50 (48 hr, mg/L)**	**Oral rat LD50 (mg/kg)**	**Bioconcentration factor**	**Solubility at 25°C (mg/L)**	**Fathead minnow LC50 (96 hr, mg/L)**	**Daphnia magna LC50 (48 hr, mg/L)**	**Oral rat LD50 (mg/kg)**	**Bioconcentration factor**	**Solubility at 25°C (mg/L)**
Diclofenac	$C_{14}H_{11}Cl_2NO_2$	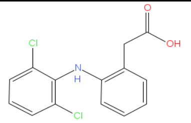	C1=CC=C(C(=C1)CC(=O)O)NC2=C(C=CC=C2Cl)Cl	0.4	3.5	244.02	26.94	5.27	0.508	0.012	0.376	0.476	0.764
DCF1 (4'-hydroxy-DCF)	$C_{14}H_{11}Cl_2NO_3$	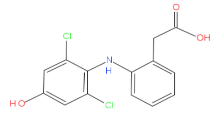	O=C(O)CC=1C=CC=CC1NC=2C(Cl)=CC(O)=CC2Cl	0.18	4.41	256.65	11.49	24.1	0.499	0.742	0.445	0.464	0.739
DCF1b (5-hydroxy-DCF)	$C_{14}H_{11}Cl_2NO_3$	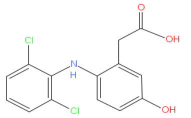	O=C(O)CC1=CC(O)=CC=C1NC=2C(Cl)=CC=CC2Cl	0.35	2.64	268	10.21	22.6	0.499	0.742	0.409	0.464	0.78
DCF3	$C_{14}H_{11}NO_2$	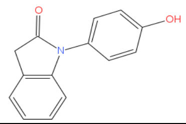	O=C1N(C2=CC=C(O)C=C2)C=3C=CC=CC3C1	2.52	9.04	604.81	31.07	35.3	0.282	0.551	0.355	0.418	0.701
Erythromycin	$C_{37}H_{67}NO_{13}$	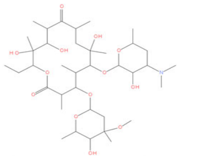	O=C1OC(CC)C(O)(C)C(O)C(C(=O)C(C)CC(O)(C)C(OC2OC(C)CC(N(C)C)C2O)C(C)C(OC3OC(C)C(O)C(OC)(C)C3)C1C)C	n/a	180.36	3097.82	n/a	387.93	n/a	0.349	0.333	n/a	0.958
ERY1	$C_{29}H_{53}NO_{10}$	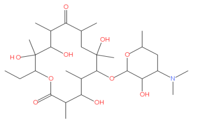	CC1C(C(C(CC)OC(C(C)C(C(C(CC(C1=O)C)(C)O)OC2C(C(CC(C)O2)[N](C)C)O)C)O)=O)(C)O)O	0.0659	303.22	2815.8	n/a	619.27	0.921	0.606	0.318	n/a	0.91
ERY2	$C_{21}H_{38}O_8$	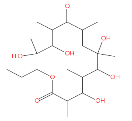	CC1C(C(C(CC)OC(C(C)C(C(C(CC(C1=O)C)(C)O)O)C)O)=O)(C)O)O	29.73	249.33	1895.94	n/a	1017.46	0.966	0.922	0.443	n/a	0.667
Gemfibrozil	$C_{15}H_{22}O_3$	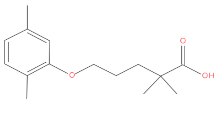	CC1=CC(=C(C=C1)C)OCCCC(C)(C)C(=O)O	3.89	5.13	469.56	16.11	17.16	0.809	0.929	0.449	0.706	0.62
GEM1	$C_{15}H_{22}O_4$	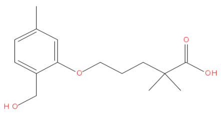	O=C(O)C(C)(C)CCCOC1=CC(=CC=C1CO)C	3.23	17.38	1966.36	5.48	193.84	0.809	0.934	0.246	0.588	0.49
GEM3	$C_{15}H_{20}O_5$	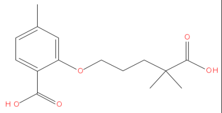	O=C(O)C1=CC=C(C=C1OCCCC(C(=O)O)(C)C)C	8.13	n/a	1652.79	1.6	211.95	0.809	n/a	0.196	0.648	0.31

LC = lethal concentration, LD = lethal dose
n/a = insufficient similar chemical data available; no predication can be made

**Fathead minnow LC50 (96 hr, mg/L)**	Aq. conc'n at which 50% of exposed fathead minnows die after 96 hours
**Daphnia magna LC50 (48 hr, mg/L)**	Aq. conc'n at which 50% of exposed D. magna (planktonic crustacean) die after 48 hrs
**Oral rat LD50 (mg/kg)**	Amt of chemical in mg/kg body weight that is lethal to 50% of rats after injestion
**Bioconcentration factor**	Ratio of the chemical conc'n in fish to that in water at steady state

**Color key:**	**MAE = Mean Absolute Error**
**MAE < 0.2**	
**MAE 0.2 - 0.4**	Mean absolute errors for similar chemicals (similarity ≥ 0.5) in the training set are reported, except when experimental values were available for the queried chemical. In that case, the MAE for similar chemicals from the test set is reported. For more details, please see Martin, T.M., et al., Toxicity Estimation Software Tool. 2020, US EPA.
**MAE 0.4 - 0.6**	
**MAE 0.6 - 0.8**	
**MAE > 0.8**	

References

1. Ren, J., et al., *Isolation and identification of a novel erythromycin-degrading fungus, Curvularia sp. RJJ-5, and its degradation pathway.* FEMS Microbiol Lett, 2021. **368**(1).
2. Llorca, M., et al., *Identification of new transformation products during enzymatic treatment of tetracycline and erythromycin antibiotics at laboratory scale by an on-line turbulent flow liquid-chromatography coupled to a high resolution mass spectrometer LTQ-Orbitrap.* Chemosphere, 2015. **119**: p. 90-98.
3. Wei, L., et al., *Transformation of erythromycin during secondary effluent soil aquifer recharging: Removal contribution and degradation path.* Journal of Environmental Sciences, 2017. **51**: p. 173-180.
4. Sathishkumar, P., et al., *Occurrence, interactive effects and ecological risk of diclofenac in environmental compartments and biota - a review.* Science of The Total Environment, 2020. **698**: p. 134057.
5. Jewell, K.S., et al., *Transformation of diclofenac in hybrid biofilm–activated sludge processes.* Water Research, 2016. **105**: p. 559-567.
6. Kosjek, T., et al., *Metabolism studies of diclofenac and clofibric acid in activated sludge bioreactors using liquid chromatography with quadrupole – time-of-flight mass spectrometry.* Journal of Hydrology, 2009. **372**(1): p. 109-117.
7. Kjeldal, H., et al., *Genomic, Proteomic, and Metabolite Characterization of Gemfibrozil-Degrading Organism Bacillus sp. GeD10.* Environmental Science & Technology, 2016. **50**(2): p. 744-755.
8. Martin, J., et al., *Multi-residue method for the analysis of pharmaceutical compounds in sewage sludge, compost and sediments by sonication-assisted extraction and LC determination.* J Sep Sci, 2010. **33**(12): p. 1760-6.
